# Mepolizumab-responsive recurrent eosinophilic eruption without peripheral eosinophilia

**DOI:** 10.1016/j.jdcr.2024.08.014

**Published:** 2024-09-02

**Authors:** Nina B. Curkovic, Irene H. Yuan, Eman Bahrani, Meagan S. Keefe, Basil M. Kahwash

**Affiliations:** a Vanderbilt University School of Medicine, Nashville, Tennessee; b Division of Allergy, Pulmonary and Critical Care Medicine, Department of Medicine, Vanderbilt University Medical Center, Nashville, Tennessee; c Department of Dermatology, Vanderbilt University Medical Center, Nashville, Tennessee; d Department of Pathology, Microbiology, and Immunology, Vanderbilt University Medical Center, Nashville, Tennessee

**Keywords:** eosinophilic cellulitis, eosinophilic folliculitis, hypereosinophilic syndrome, mepolizumab, Wells syndrome

## Introduction

Mepolizumab, an anti-interleukin (IL)-5 monoclonal antibody, is approved for use in hypereosinophilic syndrome (HES), a group of disorders characterized by peripheral eosinophilia and eosinophil-induced organ dysfunction. HES commonly presents with dermatologic manifestations.^1^ Previous case reports describe the use of mepolizumab in treating eosinophilic cellulitis (Wells syndrome), a rare cutaneous disease with dermal eosinophil infiltration, and HES-associated eosinophilic cellulitis.^2^, ^3^, ^4^ We present a case of a treatment-refractory eosinophilic eruption of several years’ duration that cleared with mepolizumab despite normal blood eosinophil levels.

## Case report

A 52-year-old male presented to allergy and dermatology clinics with a 2-year history of an exuberant pruritic eruption presumed to be chronic urticaria. The rash was first noted 2 weeks after he received the flu vaccine and spread from his head to the extremities. Initial improvement with oral corticosteroids was unsustained, and he continued to have frequent episodes of initially pruritic then painful lesions that lasted greater than 24 hours. The rash was refractory to antihistamines, mycophenolate mofetil, cyclosporine, and omalizumab. At presentation, the patient reported continued rash despite daily prednisone 40-60 mg, cetirizine 10 mg, fexofenadine 360 mg, and famotidine 40 mg.

**Fig 1:**
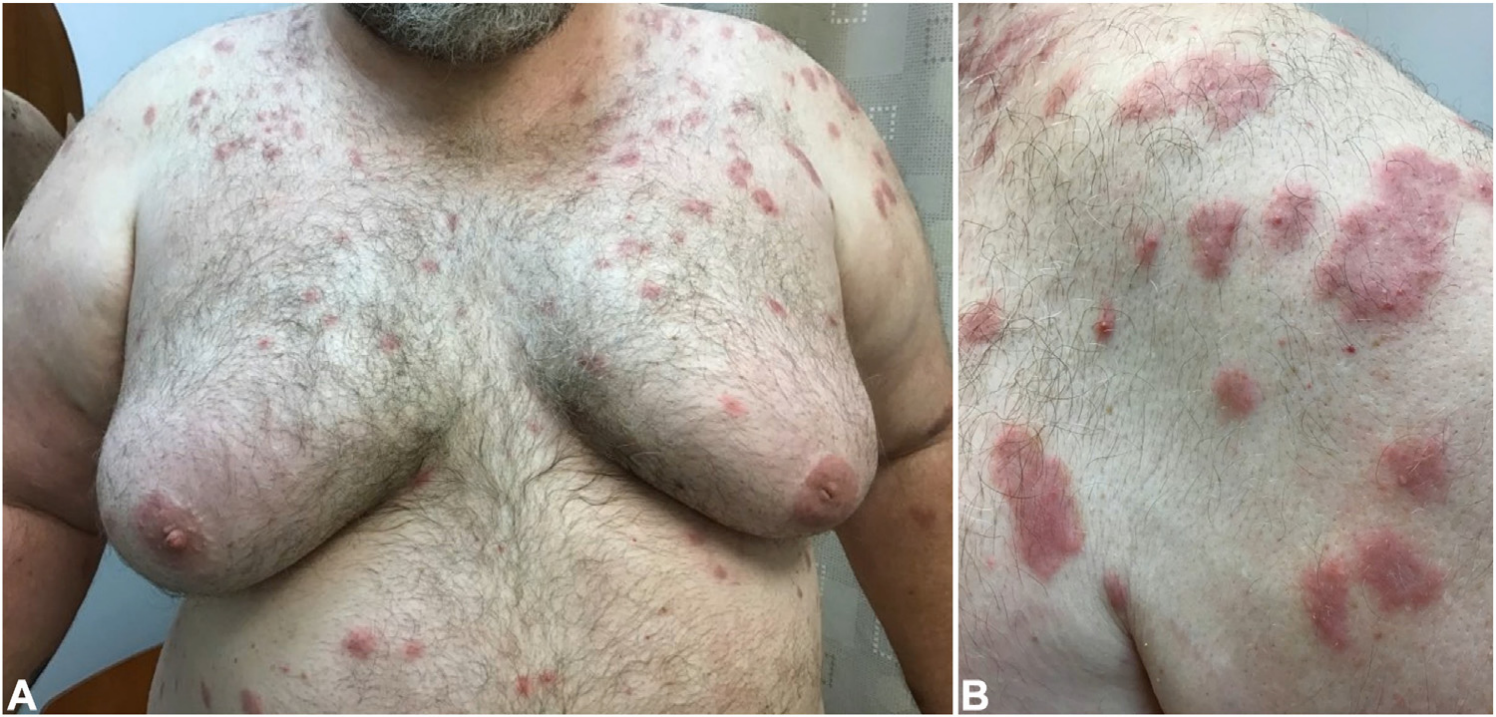
Skin findings at presentation on (**A**) chest and (**B**) closer view of the left anterolateral shoulder.

**Fig 2:**
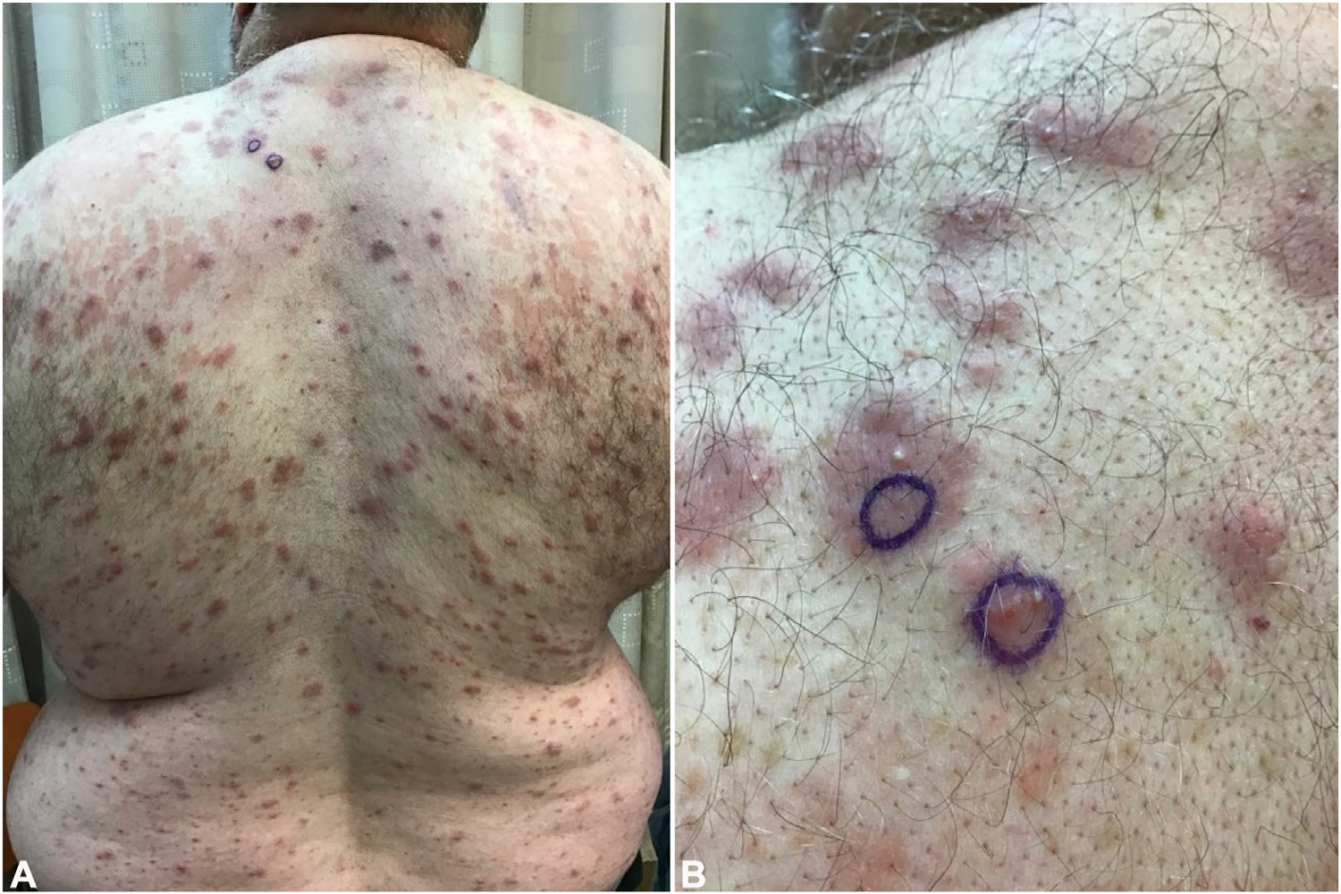
Skin findings at presentation on (**A**) back and (**B**) closer view of the left upper back.

Physical exam demonstrated intensely erythematous, edematous, and annular plaques with overlying pustules on the trunk, upper extremities, and shins (Figs 1 and 2). Initial serum tests including complete blood count with differential, comprehensive metabolic panel, thyroid-stimulating hormone, erythrocyte sedimentation rate, C-reactive protein, alpha-gal allergy panel, IgM, and IgG were unremarkable. Montelukast 10 mg daily and cyclosporine 200 mg twice daily were added to the patient’s regimen.

**Fig 3:**
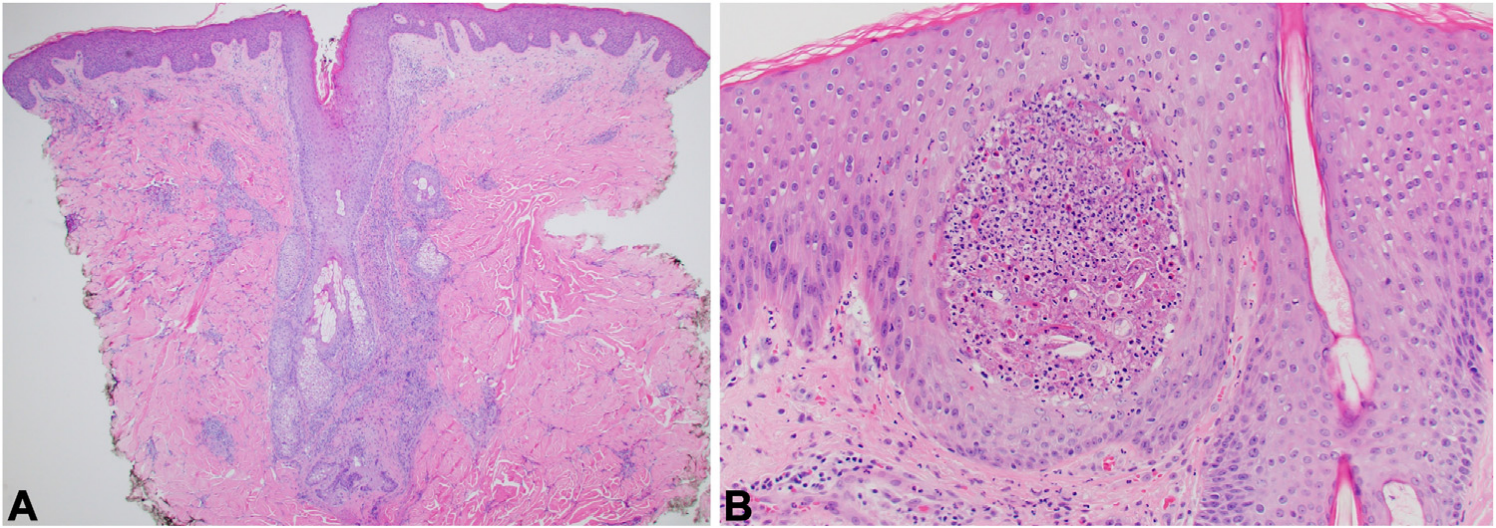
Skin histopathology demonstrating (**A**) perifollicular dermal inflammatory infiltrate (hematoxylin and eosin [H&E], 4×) and (**B**) intraepidermal eosinophilic and neutrophilic pustule adjacent to a follicle (H&E, 20×).

**Fig 4:**
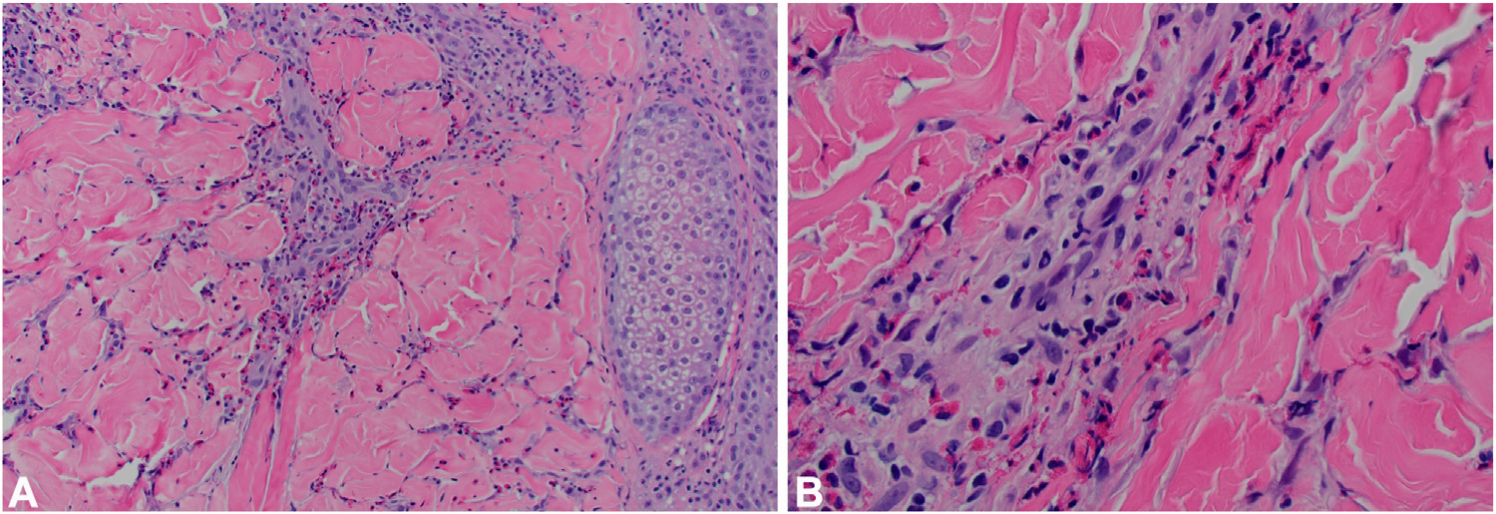
**A,** Dermal inflammatory infiltrate with a predominance of eosinophils (H&E, 20×) and (**B**) eosinophil degranulation (H&E, 60×). *H&E*, Hematoxylin and eosin.

Punch biopsies from the back and arm demonstrated perivascular and markedly perifollicular dermal inflammation with numerous eosinophils, lymphocytes, and histiocytes (Fig 3). Evidence of eosinophil degranulation, papillary dermal edema, and mild epidermal spongiosis was also noted (Fig 4). The differential included eosinophilic cellulitis, eosinophilic folliculitis, cutaneous involvement of HES, eosinophilic dermatosis of hematologic malignancy, eosinophilic annular erythema, allergic contact dermatitis, drug hypersensitivity or arthropod bite reactions, fungal or parasitic infection, a rare presentation of mycosis fungoides, and urticarial bullous pemphigoid less likely. Direct immunofluorescence, Gram stain, and fungal and bacterial cultures of a pustule were negative. Further workup for infection, including HIV, syphilis, and tick-borne disease serology, was negative.

Given the abundance of tissue eosinophils on biopsy, additional workup for HES was performed despite normal serum eosinophil counts. There was suspicion that long-term steroid use may have suppressed peripheral eosinophilia. Lymphocyte subpopulations, ANCA, IgE, and B12 levels, PDGFRA/B chromosome rearrangement, and JAK2 gene assays were unremarkable. Of note, one large clonal T-cell population not observed in biopsy specimens was detected on blood T-cell receptor gene rearrangement analysis, though the hematology team did not suspect T-cell lymphoproliferative disease or myeloid variant of HES. Of note, his peripheral eosinophil count remained normal 1 month after discontinuing steroids.

While tapering prednisone, the patient was diagnosed with peptic ulcer disease and received empiric treatment for H. pylori with clarithromycin and amoxicillin despite no confirmation of infection on gastric biopsy. He had marked improvement in his rash for several weeks while on antibiotics, during which his prednisone, montelukast, and cyclosporine were self-discontinued. Following completion of antibiotics, the patient's skin lesions reappeared. A trial of doxycycline was initiated but was ineffective. He was restarted on clarithromycin, then switched to azithromycin, which partially improved his symptoms.

Due to the refractory and eosinophilic nature of his rash, a trial of mepolizumab at 300 mg injections every 4 weeks was initiated while tapering antibiotics. At follow-up 6 months later, the patient reported complete clearance of the eruption and sustained relief for the first time in years. He remains on monthly mepolizumab, twice daily fexofenadine, and daily doxepin.

## Discussion

Dermal eosinophils on biopsy elicit a broad differential diagnosis encompassing primary eosinophilic dermatoses, parasitic infections, immunodeficiencies, hematologic malignancies, and HES. Lack of peripheral eosinophilia in the present case suggests an eosinophilic dermatosis, such as eosinophilic cellulitis (Wells syndrome) or eosinophilic folliculitis (Ofuji disease), where blood eosinophilia may be associated but is not required for diagnosis. Given that the patient's chronic steroid use could possibly have suppressed a peripheral eosinophilia, HES workup was pursued. Neither primary nor secondary causes of HES, including myeloproliferative or lymphocytic variants of HES, were identified, leaving idiopathic HES (I-HES) as a diagnostic consideration. Of note, eosinophilic cellulitis-like cutaneous findings have been reported in HES, with I-HES manifestations rarely including eosinophilic infiltration of the hair follicle and eosinophilic vasculitis.^1^, ^2^, ^3^, ^4^ Whether the presented case represents an atypical I-HES with predominantly cutaneous features or an isolated eosinophilic dermatosis remains unclear.

Interestingly, the rash improved dramatically with antibiotics. This was more notable with macrolides than tetracyclines. Tetracyclines have long been utilized in skin diseases for their anti-inflammatory properties, including in autoimmune bullous disorders, where eosinophils play a role. They can induce eosinophil apoptosis, reduce pro-inflammatory cytokine production by neutrophils, and inhibit leukocyte migration.^5^^,^^6^ Macrolides also reduce proinflammatory cytokines such as IL-6, IL-8, and tumor necrosis factor-alpha and are sometimes used as adjunct therapy in managing chronic inflammatory airway diseases like asthma, chronic obstructive pulmonary disease, and chronic rhinosinusitis^7^^,^^8^ While the eradication of H. pylori has in some cases been shown to improve chronic urticaria, suspicion for this infection was low given his lack of gastrointestinal symptoms at rash onset, unconfirmed infection prior to empiric therapy, and recurrence despite an adequate treatment course.^9^ The ultimate resolution of rash on mepolizumab suggests it appeared to respond to the anti-inflammatory rather than antibiotic effects of the agents.

To our knowledge, the utility of mepolizumab for eosinophilic dermatoses without peripheral eosinophilia has not been reported. Prior case reports of eosinophilic dermatoses treated with mepolizumab all featured peripheral eosinophilia, and one case report of chronic spontaneous urticaria treated with mepolizumab was in a patient with eosinophilic asthma.^2^, ^3^, ^4^^,^^10^ We report the successful treatment of an unspecified eosinophilic dermatosis with mepolizumab, highlighting a potential role for anti-IL-5 biologics in eosinophil-driven skin diseases, regardless of a peripheral eosinophilia background.

## Conflicts of interest

Dr Kahwash receives research support from 10.13039/100004330GlaxoSmithKline. Drs Curkovic, Yuan, Bahrani, and Keefe have no conflicts of interest to declare.
